# The Microbial Community of a Terrestrial Anoxic Inter-Tidal Zone: A Model for Laboratory-Based Studies of Potentially Habitable Ancient Lacustrine Systems on Mars

**DOI:** 10.3390/microorganisms6030061

**Published:** 2018-06-30

**Authors:** Elliot Curtis-Harper, Victoria K. Pearson, Stephen Summers, John C. Bridges, Susanne P. Schwenzer, Karen Olsson-Francis

**Affiliations:** 1 Faculty of Science, Technology, Engineering and Mathematics, The Open University, Walton Hall, Milton Keynes MK7 6AA, UK; ecurtisharper@gmail.com (E.C.-H.); v.k.pearson@open.ac.uk (V.K.P.); susanne.schwenzer@open.ac.uk (S.P.S.); 2 Singapore Centre for Environmental Life Sciences Engineering, Nanyang Technological University, 60 Nanyang Drive, 637551 Singapore, Singapore; ssummers@ntu.edu.sg; 3 Space Research Centre, Department of Physics and Astronomy, University of Leicester, Leicester LE1 7RH, UK; j.bridges@le.ac.uk

**Keywords:** Mars, lacustrine system, habitability, analogue community

## Abstract

Evidence indicates that Gale crater on Mars harboured a fluvio-lacustrine environment that was subjected to physio-chemical variations such as changes in redox conditions and evaporation with salinity changes, over time. Microbial communities from terrestrial environmental analogues sites are important for studying such potential habitability environments on early Mars, especially in laboratory-based simulation experiments. Traditionally, such studies have predominantly focused on microorganisms from extreme terrestrial environments. These are applicable to a range of Martian environments; however, they lack relevance to the lacustrine systems. In this study, we characterise an anoxic inter-tidal zone as a terrestrial analogue for the Gale crater lake system according to its chemical and physical properties, and its microbiological community. The sub-surface inter-tidal environment of the River Dee estuary, United Kingdom (53°21′15.40″ N, 3°10′24.95″ W) was selected and compared with available data from Early Hesperian-time Gale crater, and temperature, redox, and pH were similar. Compared to subsurface ‘groundwater’-type fluids invoked for the Gale subsurface, salinity was higher at the River Dee site, which are more comparable to increases in salinity that likely occurred as the Gale crater lake evolved. Similarities in clay abundance indicated similar access to, specifically, the bio-essential elements Mg, Fe and K. The River Dee microbial community consisted of taxa that were known to have members that could utilise chemolithoautotrophic and chemoorganoheterotrophic metabolism and such a mixed metabolic capability would potentially have been feasible on Mars. Microorganisms isolated from the site were able to grow under environment conditions that, based on mineralogical data, were similar to that of the Gale crater’s aqueous environment at Yellowknife Bay. Thus, the results from this study suggest that the microbial community from an anoxic inter-tidal zone is a plausible terrestrial analogue for studying habitability of fluvio-lacustrine systems on early Mars, using laboratory-based simulation experiments.

## 1. Introduction

The surface of present day Mars is deemed inhospitable to life. The environment is cold, dry, highly oxidised and exposed to ultraviolet (UV) and ionizing radiation. On early Mars, the surface conditions appear to have been more conducive to life, with a warmer climate and a denser atmosphere that could provide protection from UV and cosmic radiation [1,2,3,4]. Globally and locally, ancient fluvial systems, of various orders of magnitude, are observed, which may have once been habitable (e.g., [5,6,7,8,9]). Sediments formed by those processes preserve evidence of those ancient conditions (and potential biomarkers within), so they can be studied to decipher the past. Unambiguous, ground-based evidence of a range of past aqueous activity has been collected from Gale crater by the Mars Science Laboratory rover Curiosity since 2012 (e.g., [8,10,11,12,13]). Thus, we chose this example to compare—and as necessary contrast—our analogue site. It must be noted that there is also ubiquitous evidence for similar systems elsewhere on Mars [14].

### 1.1. Geology and Lake Evolution at Gale Crater

Gale crater is a 155 km diameter impact crater at the border of the ancient highlands and Elysium Planitia (4.491 S, 137.421 E). It formed during the late Noachian, creating impact melt, an ejecta blanket and a central uplift [15], all of which are currently covered by sediment and/or eroded. Before the arrival of the Curiosity rover, the crater had been extensively studied with remote data, including evidence for the existence of a fluvio-lacustrine system [10,14] the sedimentology of the central mount (e.g., [16]) and the occurrence of sulphate-, haematite-, and clay-bearing signatures in CRISM spectra [16,17].

After landing, mineralogical studies from the Curiosity rover have been used to estimate characteristics of Gale crater’s aqueous environment at Yellowknife Bay, indicating circumneutral pH, temperatures suitable for long-standing water, and salinity of between 1 and 2%. For example, the Sheepbed mudstone, discovered at Yellowknife Bay, gives strong indications of circumneutral fluid activity (due to the prevalence of clay minerals), sediment transport and low salinity [10,18,19]. These data have been used to ascertain and interpret our contemporary understanding of potentially habitable fluvio-lacustrine systems on ancient Mars, as well as understanding the presence of bio-essential minerals and carbon sources [10,20,21]. In summary, Gale’s early clay-formation history has been deduced to have been circumneutral, with potentially varied, diagenetic redox conditions (e.g., [10,19,22,23,24]). Oxychlorine phases (chlorates/perchlorates) have been detected in varying amounts in the Gale sediments, ranging from as little as 0.05 ± 0.025 to a maximum of 1.05 ± 0.44 wt.% ClO_4_ [21], and alongside with iron sulphides, and the SAM-EGA evolution of H_2_S attest to a variety of redox conditions in the rocks [18,21]. Despite these redox agents, organic carbon and nitrogen have been detected and support the concept of habitable conditions [10,21]. Most recently, the preservation of organic carbon, aided by the formation of organic sulphur molecules, has been reported in the lacustrine mudstones at the base of the lake sediment sequence [25].

With ongoing mission progress upwards in stratigraphy, a much more varied geological history has been found; most important for this study are sandstones, silica-rich deposits, and the fact that Gale shows signs of repeated evaporation to dryness. Yellowknife Bay strata are mud- and siltstones, but coarser fractions have been found early in the mission, with the detection of conglomerates indicating a fluvial-lacustrine-deltaic system with minor dry episodes, all comprising the Bradbury group (e.g., [8,10,12]). Stratigraphically higher is the Mount Sharp group, which includes the Murray mudstone-dominated unit. Overlying both units is the Stimson sandstone, which formed in a dry, aeolian setting, thus marking a dramatic transition of conditions [26]. The fluvio-lacustrine sediments experienced circumneutral diagenesis at up to about 50 °C (e.g., [11,19]) during burial of the sediments, forming a clay and Fe oxide bearing secondary assemblage [18]. However, in localised areas some jarosite and evidence of element mobility associated with intense leaching have been taken as indicative of alteration in acidic conditions (e.g., [24,27]). Silica-rich deposits have also been detected and are thought to be the result of tridymite-rich detrital sediments [28], which in places have undergone alteration and silica remobilisation along fractures at low temperatures [29].

In addition, evidence for evaporation, occasionally to dryness, has been found chemically, geomorphologically and through hydrologic modelling (e.g., [12,30,31]). A visual example of this was the discovery of mud cracks in the Murray formation [32]. This short summary demonstrates how the fluid conditions are thought to have changed over time, both episodically and towards the dry conditions observed today, from circumneutral to acidic, and from low to high salinity, and with varying redox [19,22,27,30,32,33]. Higher-salinity fluids, analogous to those found in the terrestrial marine environment, have thus occurred repeatedly.

**Figure 1:**
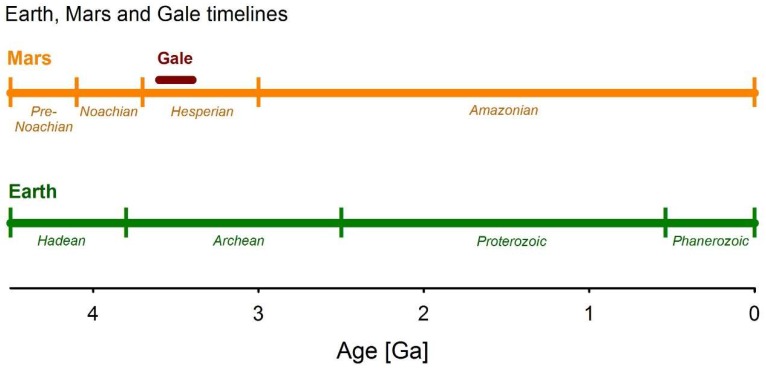
Timeline of the geologic eons on Earth and Mars, drawn after Carr and Head (2010) [44], see there for details of the geologic history of Mars. The red bar shows the timing and duration of the sedimentary cycle at Gale crater as given by Paulucis et al. (2016) [34].

Throughout the early Hesperian time period (Figure 1), it is postulated that the Gale crater lacustrine system underwent episodic drying periods, with intermittent lakes present over approximately 30,000 Earth years [34]; however, the absolute timing of the sedimentation cycle is currently uncertain. It has been suggested that regional groundwater flow contributed significant amounts of water to Gale crater [34], and thus may have provided a refuge for microbial life when surface, and shallow subsurface, desiccation occurred. The conditions associated with Gale crater sediments are probably not unique. Circumneutral pH conditions have been inferred for other locations on Mars, for example the Terby and Jezero craters [35,36,37], and evidence of acid alteration and evaporation have been found and modelled (e.g., [22,38,39,40,41]). Since Gale crater is the target of current *in-situ* analyses, and future Mars missions are likely to target similar fluvial or lacustrine environments (see, e.g., [42,43]), identifying and characterising the microbial communities that could be supported by lacustrine systems is of fundamental importance to determining potential habitability on past and present Mars. Such studies rely on terrestrial analogues that match or simulate Martian environmental conditions and/or utilise microbial communities that can survive within these conditions.

### 1.2. Microbes in Analogue Systems

Investigating the microbial community within an analogous active system can give insights into the microbial processes that could occur and any resulting bio-signatures that could be used as evidence of life. Traditionally, such studies have predominantly focused on microorganisms from extreme terrestrial environments, such as those with high salinity, cold temperatures and high acidity, or microorganisms that are chemolithoautotrophic [45,46,47,48,49]. These are applicable to a range of Martian environments, for example the acidic environments indicated by the presence of jarosite at Meridiani Planum; however, they have limited relevance to the lacustrine systems evident at Gale crater [50,51]. Only limited work, to date, has utilised terrestrial analogues for lacustrine systems; this has focused on microorganisms from general environmental comparisons (e.g., fluvial-lacustrine environments in Iceland [52], direct lithological comparisons (e.g., the Green River Formation [53]), or mineral specific analogues (e.g., a clay mineral analogue of Yellowknife Bay [54]). Since the detection of calcium sulphate veins on Mars, there have been several terrestrial analogue environments proposed that mimic the geochemical processes that occurred at Gale crater. These analogues include such diverse environments as the Upper Triassic mudstones at Watchett Bay, UK [30] and the Triassic Moenkoepi Formation mudstones in Utah, USA [55]. The formation of sulphate veins at these sites has been proposed to be analogous to the formation of similar mineralogies in the clay sediments at Gale crater, but no attention was given to those sites’ local microbial communities.

### 1.3. Comparing and Contrasting the Analogue Site Prerequisites to Gale Crater

Here we propose the microbial community from the anoxic inter-tidal zones as analogues for the ancient lake system at Gale crater, and other equivalent locations on Mars, which we chose on the basis of the following expected similarities: an estuary environment has a salinity of between 0.5 and 3.5%, which is comparable to the 1–2% salinity proposed for the ancient Martian lake system [11,18,56]. The pH is approximately circumneutral [56], which is comparable to the pH of the proposed aqueous environment at Gale crater [19,57]. Inter-tidal zones are also subjected to periods of drying; daily drying occurs within the upper region of the intertidal sediments and long-term drying occurs within the backshore. These periods of exposure to air can also result in changes to the salinity of this environment; drying raises salinity but precipitation causes salinity to lower to below that of a typical marine environment. Morphological evidence suggests that most drying episodes were longer in the Gale crater lacustrine system with periods of drying many orders of magnitude longer (potentially thousands of years versus diurnal) than seen at the River Dee site chosen for this study, but desiccation cracks at the target ‘Old Soaker’ also record rapid changing lake levels with the associated local changes in conditions [32]. In addition, our focus was the region below the Redox Potential Discontinuity (RPD), which is anoxic and might have useful analogies to the moderately oxidising environment indicated by Gale crater sediments [58].

The environmental conditions within the analogue site at the River Dee estuary, UK were characterised and compared to the conditions known at Gale crater. In parallel, the microbial community was identified as an analogue for putative life, which could be used in laboratory-based simulation experiments to study microbial processes in ancient Martian lacustrine systems. In this way we can identify biological processes, and subsequent bio-signatures that could have existed in ancient lacustrine systems on Mars.

## 2. Materials and Methods

### 2.1. Sample Site and Sample Collection

**Figure 2:**
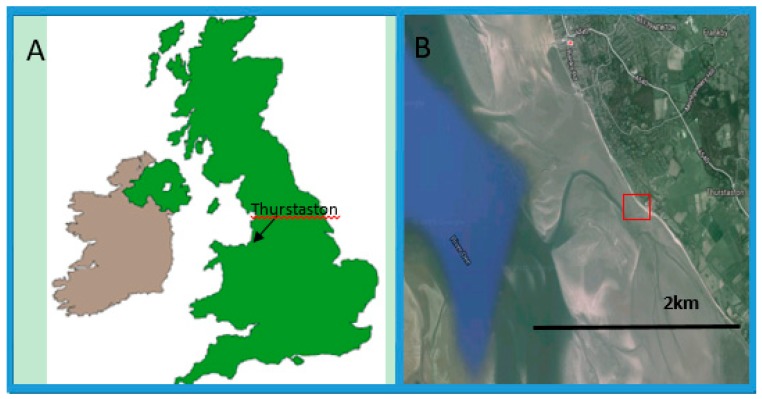
(**A**) Map of the United Kingdom with an arrow showing the location of the sample site; (**B**) an image of the River Dee estuary. The red box denotes the sampling site location that was used in this study [61].

The site for this study was the Thurstaton region of the River Dee estuary, UK (53°21′15.40″ N, 3°10′24.95″ W) (Figure 2). The site is situated approximately 8 km from the mouth of the estuary. At this location, the salinity of the water is approximately 2.6% (26 ppm) [59]. The estuary is believed to have been glacially cut and has evolved through erosional processes over 18,000 years, resulting in a relatively large basin for the volume of river water discharged [60].

In November 2013, sediment samples were aseptically collected from 30 cm below the surface and beneath the RDP Layer, where the conditions were anoxic (Eh value of approximately −156 mV). The sample sites were determined using a 30 × 40 m grid layout, which was located 100 m from the water at low tide (at high tide the site was submerged). The sample grid consisted of two rows 10 m apart that ran parallel to the estuary, with four sample sites on each row at 10 m intervals. Sites were numbered as follows: 1 to 4 in row one (nearest the water) and 5 to 9 in row 2.

All tools used to collect samples were cleaned with 95% ethanol and then rinsed with autoclaved ddH_2_O between sampling. Approximately 10 g of sediment was collected, aseptically, from each site and stored in a plastic Whirl-Pak bag. The samples were kept at ambient temperature during field work and transported at 4 °C (for approximately four hours). On return to the laboratory, sub-samples (2 g) were stored at −80 °C for nucleic acid analysis; whilst the remaining sample was stored at 4 °C, for further analyses.

### 2.2. Temperature, pH and TOC

Temperature and pH measurements were carried out at the estuary in situ. Temperature was measured using an RS 1327 K Infrared Thermometer. The probe was inserted into the sample, below the RDP zone, taking care to avoid any areas that were in, or had been in, direct sunlight. The probe was left in place until a steady reading was obtained (accuracy of ±0.02). For pH measurements, a Thermo Scientific (Waltham, MA, USA) Orion Three Star pH probe (±0.002 pH unit accuracy) was used, which was calibrated using Omega Buffer solutions at pH 4 and 10. The probe was inserted below the RDP zone and left in place until a steady reading was obtained.

Total Organic Carbon (TOC) measurements of the sediment samples were carried out using a Total Carbon Analyser (TOC-V C5N) with an SSM-5000A solid state module (Shimadzu, Kyoto, Japan). One gram of wet weight sediment (*n = 3*) was dried for 2 h at 180 °C prior to analysis. For calibration, a glucose standard was used, as recommended by the manufacturer.

### 2.3. Mineralogy

A polished block was produced by fixing a pellet of compressed sand in epoxy-resin and polishing the surface. This block was carbon-coated using a K950X Turbo carbon sputter coater (EMITECH, Montigny-le-Bretonneux, France). The mineralogy of the sand was examined using an FEI Quanta 3D dual beam scanning electron microscope with a 80 mm X-MAX energy dispersive X-ray detector in energy dispersive spectroscopy (EDS) mode (Oxford Instruments, Oxford, UK). Elemental mapping was carried out using an acceleration voltage of 20 kV and a beam current of 0.6 nA. Point spectra were taken at discrete locations on each sample. The presence of Si, Na, K, Al, P, S, Cr, Fe, Ti, Mg, Ca and Cl was mapped to facilitate mineral identification, with proportions of each mineral calculated using ImageJ software (https://imagej.nih.gov/ij/).

### 2.4. Cell Enumeration

Total cell numbers were determined by adding 1 g (wet weight) of sample to 1 mL of sterilised ddH_2_O. The samples were stained with Sybr Green DNA stain, and analysed using a Leica DMRP microscope equipped with epifluorescence (Leica Microsystem, Bensheim, Germany), as previous described [60]. All enumerations were conducted with 50 fields of view counted per sample.

### 2.5. Microbial Community Analysis

The microbial communities were fingerprinted by terminal restriction fragment length polymorphism (tRFLP) analysis of the 16S rRNA gene. Genomic DNA was extracted from 0.5 g (wet weight) of sediment using the phenol/chloroform extraction protocol previously described [62]. The 16S rRNA gene was partially amplified for bacteria and archaea using the following primers: 6FAM labelled 63f and 530r, and 6FAM labelled A341f and A1204r [63,64,65].

Amplifications were carried out identically using 10 ng template DNA, 250 nM of each primer, 5 μL of 10 × Taq buffer, 2 mM MgCl_2_, 0.1 mM of each Deoxynucleotide Triphosphate (dNTP), 5 μg bovine serum albumin (BSA) and 1.75 U of Taq. PCR conditions were as follows for the bacteria: initial denaturation at 94 °C for 10 min. This was followed by 35 cycles of: denaturing 45 s 94 °C, annealing 1 min at 56 °C, elongation 3 min 72 °C. Final elongation was for 10 min at 72 °C. For archaea, the conditions were as follows: initial denaturation at 94 °C for five min, followed by 35 cycles of denaturing for one min at 94 °C, annealing at 55 °C for one min and extension at 72 °C for one min, followed by a final extension for ten min.

The amplified gene fragments were purified using a Qiagen PCR purification kit according to manufacturer’s guidelines. Concentrations of purified PCR products were quantified using a ThermoScientific NanoDrop 100 spectrophotometer, according to manufacturer’s guidelines. Purified PCR products (10 ng) were digested in mixtures containing 10 U of MspI restriction endonuclease, 0.1 μg of BSA and 1 μL of CutSmart enzyme buffer (×10) and made up to 10 μL using sdH_2_O. The digestion was carried out at 37 °C overnight and analysed using a 3730 Sanger sequencer (Macrogen, Seoul, Korea). The resulting electropherograms were analysed with SoftID Genemarker V2.6.4 software (SoftGenetics, State College, PA, USA). Quality filtering was carried out and tRFs smaller than 100 bp and with an intensity of <50 units were not included.

To identify the approximate taxonomy of the individual peaks, in-silico digestions were carried out on MiSeq sequencing from site 3 using the 28f and 530r primers for bacteria, and the 340f and 534r for archaea sequences. The MiSeq sequencing was carried out at a commercial sequencing core facility (Research and Testing Laboratory, Lubbock, TX, USA). The sequences were analysed using the QIIME pipeline [66]. Briefly, the sequences were filtered and those with <300 nucleotides were excluded. The chimeric sequences were checked for using ChimeraSlayer [67]. Classification was carried out by aligning the sequences against the Silva sequence database [68] and taxonomy generated using the Naïve Bayesain rRNA Classifier version 1.0 tool within the Ribsomal Database Project classifier [69]. All sequences were submitted to NCBI under the accession numbers: SAMN07176080 (archaea) and SAMN07176079 (bacteria).

In-silico digestions of the FASTA formatted sequences were carried out by trimming the MiSeq sequences at the tRFLP primer binding sites as well as the *MspI* cleavage site. The resulting sequence fragment lengths were compared with those obtained from tRFLP data (http://nebc.nerc.ac.uk/cgi-bin/trflp0_2.cgi). Each individual peak was assigned a taxon where >75% of the in-silico terminal restriction fragments (tRFs) from the MiSeq sequence matched de novo tRF length.

### 2.6. Isolation of Microorganisms

The isolation of anaerobic microorganisms was carried out using a marine-based medium containing (g L^−1^): 9.45 of NaCl, 8.8 of MgCl_2_; 5 of Peptone; 3.24 of NaSO_3_; 1.8 of CaCl_2_; 1 of yeast extract; 0.55 of KCl; 0.16 of NaHCO_3_; 0.10 of ferric citrate; 0.02 of H_3_BO_3_ and 0.008 of K_2_HPO_4_. The pH was adjusted to 7 with 1 M HCl and aliquoted into hungate tubes prior to autoclaving. The media was inoculated aseptically under anaerobic conditions with 1 g of sample, and then incubating at 14 °C. After 14 days, the cultures were examined using the Leica DNRP microscope at 100× magnification [62]. Repeated serial dilutions were carried out to isolate microorganisms from the enrichments.

The bacteria were identified based on near-full length 16S rRNA gene sequences. Total nucleic acids were extracted from the isolates using the Phe:Chl:Iaa bead beating protocol described by [62]. The 16S rRNA gene was amplified using two sets of primers: 27f-Com2 and Com1-1541r, as previously described [62]. BioEdit software (version 7.1.3.0, Ibis Therapeutics, Carlsbad, CA, USA) was used to align the sequences and the resulting contigs were approximately 1500 bp in length. The nearest sequences were identified in the GenBank database using the BLASTN program. All contiguous sequences were deposited into Genbank.

### 2.7. Microbial Growth Experiments

Growth experiments were conducted with the isolates under conditions associated with fluvio-lacustrine systems on early Mars. The isolates were grown under anaerobic conditions, using a Mars simulation gas as the headspace (95.3% carbon dioxide, 2.7% nitrogen, 1.7% argon, 0.2% oxygen and 0.03% water vapour), 15 °C, circumneutral pH and 2% salinity. For these experiments, a minimal medium was used, which contained (g L^−1^): 1 of NH_4_Cl, 2 of Na-Lactate, 1 of Na-thioglycollate, 1 of ascorbic acid, 37 g NaCl and 13.25 g Na_2_CO_3_.

To monitor microbial growth and pH, 1 mL aliquots were aseptically removed after 1, 4, 7, 14, 21, 28 days. Cells were stained with the nucleic acid-binding dye SYBR Green I DNA (0.1% *w*/*v* stock; Life Technologies, Paisley, UK). One mL of culture was filtered through a 0.2 μm black polycarbonate filter and then washed with 100 μL of dd H_2_O. Cells were enumerated using a Leica DMRP microscope equipped with epifluorescence, as previously described [62].

### 2.8. Statistical Analyse

Statistical analyses were carried out using the Primer E (version 5), R stat version 3.2.4 and Microsoft Excel 2016 software packages [70,71]. The dissimilarity of tRFs between each of the sample rows (distance from the water), and within rows, were tested using an analysis of similarity (ANOSIM) within Primer 5 [70].

Visualisation of the individual peak dissimilarity was carried out by constructing a Principal Component Analysis (PCA) plot. Measures of tRF diversity were obtained by calculating the Shannon–Weiner diversity index (H’), although attributing diversity indices to community fingerprints introduces an inherent error [72]. To determine which tRFs had the greatest influence on differences between bacterial communities, a SIMPER analysis [73] was carried out. To visualize the operational taxonomic unit (OTU) richness of each sample, a rarefaction curve was generated using the R software package. Any differences in OTU richness evident from rarefaction were confirmed by a Mann–Whitney *U*-test of difference.

One-way analysis of variance (ANOVA) was used to investigate the difference between sample sites with respect to their environmental characteristics.

## 3. Results

### 3.1. Temperature, pH and TOC

There was no significant difference between temperature, pH values and TOC between each sample site, when examined using parametric analyses (ANOVA) (Table 1). Therefore, mean values were determined for each parameter: 12.4 ± 0.7 °C; pH 8.2 ± 0.1; TOC 1.29 ± 0.3%.

### 3.2. Mineralogy

**Figure 3:**
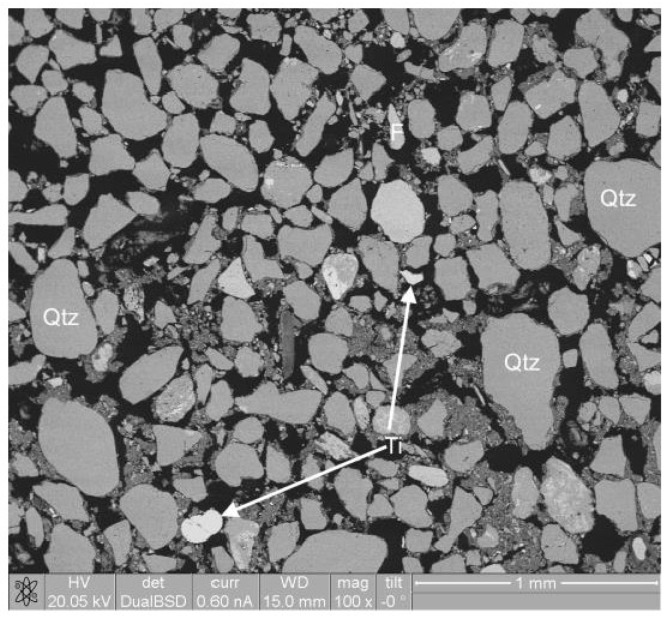
Backscattered electron images of the River Dee sediment, dominated by quartz (Qtz) grains. Minor feldspar (F) was evident. Higher contrast areas correspond with high atomic weight elements, and were confirmed to be titanomagnetite (Ti).

**Figure 4:**
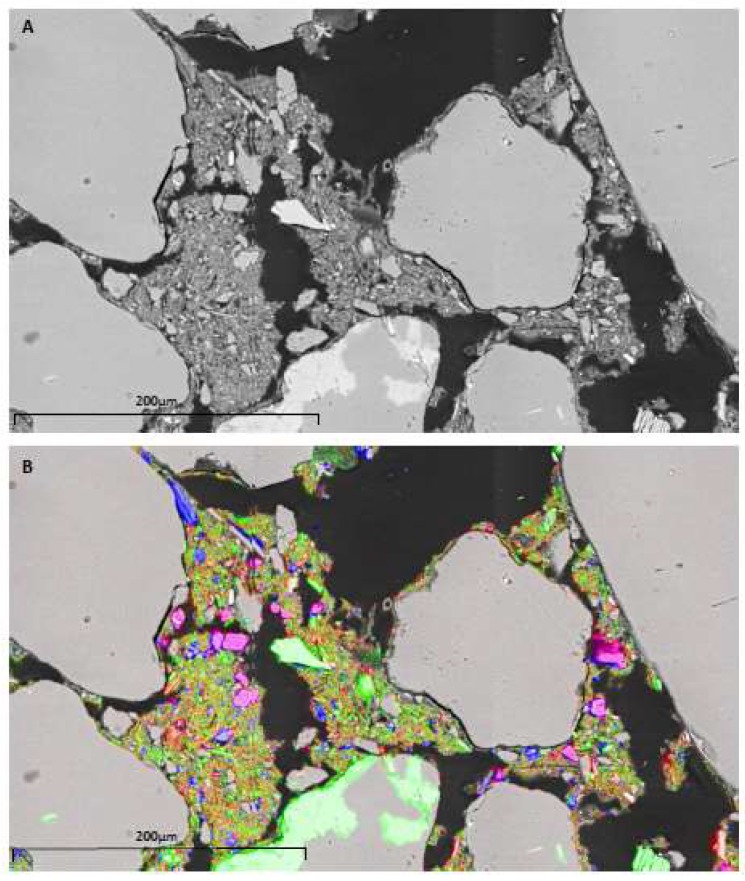
(**A**) Backscatter electron image of River Dee sediments, with detrital material surrounding quartz grain boundaries; (**B**) false colour map indicating the distribution of Mg (red), K (blue) and Ca (green).

SEM analyses showed that the dominant phase was quartz (Figure 3), present as rounded to sub-rounded grains. Other phases included feldspars (potassium-rich, likely to be sanidine), titanohaematite and apatite. Several grains were surrounded by fine-grained detrital material (Figure 4) with a composition analogous to phyllosilicate (clay) minerals, most likely illite. Calculations of modal mineralogy indicated the sediments consisted of: 63% quartz, 23% clay, 9% sanidine and 5% minor fractions.

### 3.3. Cell Enumeration

There were no statistically significant differences between sites within or between rows (*p* > 0.1) (ANOVA). The mean value for row 1 was 3.53 ± 1.2 × 10^8^ cells per gram and row 3 was 2.5 ± 0.9 × 10^8^ cells per gram; the overall mean for all sites was calculated to be 3.3 ± 1.3 × 10^8^ cells per gram.

### 3.4. Community Analysis

**Figure 5:**
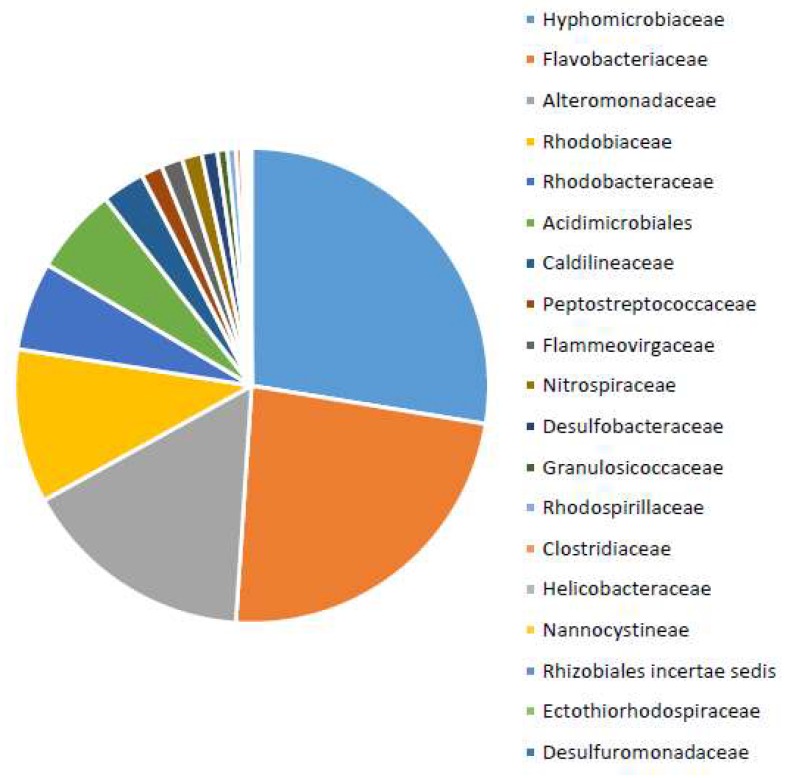
Relative abundance of the common bacterial taxa from the anoxic intertidal zone of the River Dee estuary. Data obtained from 16S rRNA gene sequences.

The microbial communities within the sample sites were examined using a combination of MiSeq and community fingerprinting. The sample site with the greatest variation in the individual peaks was selected for MiSeq data. The partial 16S rRNA gene sequences obtained with MiSeq resulted in a total of 5134 raw reads, which, after quality filtering, resulted in 1031 sequences. The bacterial community was dominated by the families *Hyphomicrobiaceae* (28%), *Flavobacteriaceae* (23%) and *Alteromonadaceae* (16%), making up 67% of the community, as shown in Figure 5.

**Figure 6:**
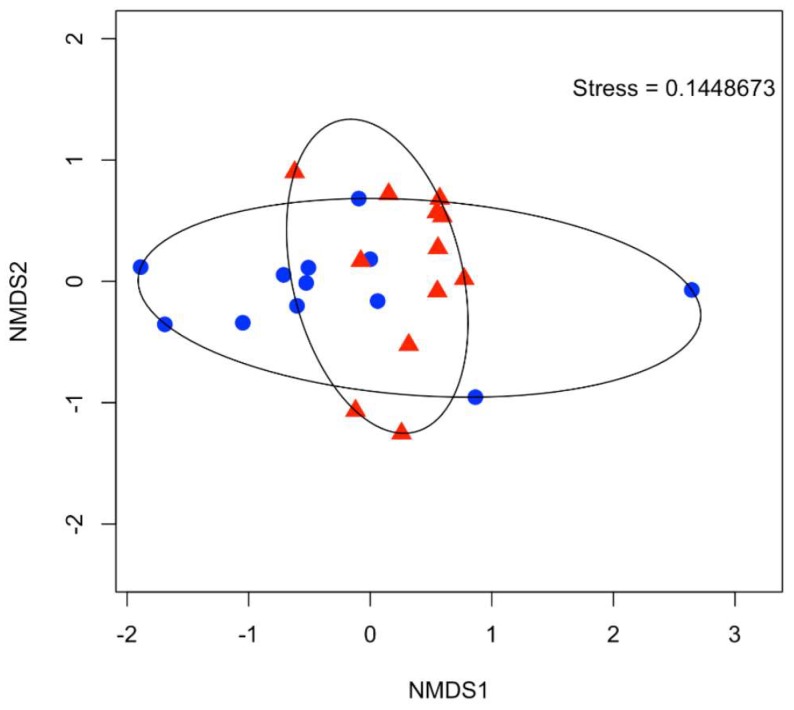
NMDS ordination demonstrating the similarities in the bacterial community between each of the sites using tRFLP analysis. Sites 1–4 are highlight in blue and sites 5–8 are highlighted in red.

To examine variation in relative abundance within the sample site, the communities were examined by community fingerprinting. Based on the in-silico digestion, each tRF was taxonomically classified (Table S1). There was no significant difference in the relative abundance between the taxonomic groups at each of the sites. PCA analysis demonstrated that there was no obvious difference between the sites (Figure 6). Examination of the α-diversity (Shannon–Weiner; H’) also confirmed that there was no difference between the sites.

For archaea, sequences obtained resulted in a total of 7163 raw reads, which, after quality filtering, resulted in 6386 sequences. The community was dominated by *Thaumarchaeota* phylum, resolved down to the *Nitrosopumilus* genus. This was the only sequence with above 80% confidence. The tRFLP data for each of the samples sites demonstrated that the *Nitrosopumilus* genus was dominant in all of the samples (~65%). There was no significant difference between sites (ANOSIM, *R* = −0.13 (*p* > 0.5).

### 3.5. Microbial Isolates

From the anoxic sediment three anaerobic isolates were obtained, which belonged to the bacterial classes Bacilli, Clostridia and Gamma-proteobacteria, as shown in Table 2. The Bacilli isolates showed 99% identity to a cultivated strain of the genus Bacillus (E01). The Clostridia showed 98% similar identity to the genus Clostriuduim (E02); the Gamma-proteobacteria showed 99% similarity to the genus Acinetobacteria (E01).

Each of the isolated were screened for their ability to grow under environmental conditions associated with the ancient lacustrine environment at Gale crater (15 °C, pH 7, salinity of 2%, and a Mars simulation gas headspace). The specific growth rates were determined as 0.015 h^−1^, 0.24^−1^ and 0.17^−1^ for E01, E02 and E03, respectively.

## 4. Discussion

### 4.1. An Analogue for the Fluvio-Lacustrine System at Gale Crater?

Evidence from recent Mars missions has demonstrated the heterogeneity of Martian environments across geological time and geographical location (e.g., [2,58,74,75,76,77]). Thus, the focus of this study was the well-characterised, and potentially habitable, fluvio-lacustrine system at Gale crater (see Section 1.1). As shown above, results from Curiosity rover suggest that the conditions in Gale crater were not as “extreme” as traditional Martian analogue environments, such as Rio Tinto or the Atacama Desert, have been proposed to mimic. Instead, temperatures may have been suitable for long-standing surface water with circumneutral pH, clay-formation, locally deviating host rock chemistry, and 1–2% salinity. These conditions do not adequately compare to existing inland or fully marine analogue environments but do match estuarine environments such as the one proposed here.

The focus of this study was, specifically, the anaerobic zone below the RDP where reducing conditions dominate. This was chosen to be comparable with early Mars’ atmosphere, which is likely to have been oxygen poor [78], resulting in anoxic (or even anaerobic) conditions beneath the planet’s surface. Further, evidence from Curiosity’s CheMin indicates that the mudstones of the Sheepbed Formation at the John Klein (JK) and Cumberland drill holes were deposited under only moderately oxidising conditions [10,18,79]. For instance, the presence of saponites indicates that Fe^2+^ oxidation was limited, avoiding the very low pH associated with widespread acidic conditions [58]. Hence, the low-oxygen conditions of the River Dee estuary provide an analogy with those of Gale crater’s lacustrine system.

The mean total organic carbon (TOC) content of the River Dee sites was calculated to be 1.29 ± 0.3%. This is higher than at Gale crater, where preliminary estimates, based on evolved CO and CO_2_ measured Curiosity’s SAM instrument, of organic carbon abundance in Gale crater sediments of between 0.08 and 0.24% [80]. Chlorinated hydrocarbons, detected by Curiosity’s SAM, GC-MS instruments [81], are believed to be, in part, reaction products from the combustion of chlorinated derivatisation products carried in the rover’s sample acquisition and analysis system [82,83]. Other carbon sources are required to account for the quantity of CO_2_ detected from the John Klein mudstones, for example the decomposition of Fe/Mg-carbonates [20], a low-abundance indigenous organic component or exogenous meteoritic organic carbon [40], but not have been positively identified.

Organic carbon has been identified in Martian meteorites [84,85], and analyses suggest this may be derived from an influx of organic material to the Martian surface from carbonaceous meteorites. McLennan [85] proposed that an influx of such material could provide between 0.03 and 0.12% organic carbon, in line with the preliminary calculations of Sutter et al. [80], and most recent observations have found a rich diversity of organic species in the Pahrump Hill mudstones [25]. Until unequivocal evidence for the existence of indigenous organic species is confirmed, however, Gale crater remains an organic carbon-limiting environment.

Despite this, Gale crater may not have always been an organic carbon-limiting environment. Evidence for oxidative post-depositional processing in the sediments [82] and the harsh radiation environment at the surface could have degraded any indigenous organics. Therefore, it is possible that the estimates of organic carbon at Gale crater are conservative, and there could have been sufficient organic carbon present on early Mars to support heterotrophic microbial communities. The River Dee site would therefore offer a plausible analogue environment with respect to organic carbon abundances.

One key difference between the analogue site and the majority of sediments at the ancient lacustrine environment in Gale crater is the bulk phase mineralogy. The samples from the River Dee estuary contained 63% quartz; Table 2 shows for Gale crater that the mineralogy of John Klein and Cumberland drill sites, which are representative of altered basaltic host rock compositions, were instead dominated by plagioclase feldspar. However, at Buckskin a rock with 40% SiO_2_-phases (tridymite and cristobalite polymorphs) has been observed, with additional SiO_2_ contained in the amorphous phase. This site contains ~75% SiO_2_, the River Dee site about 80%. Quartz and SiO_2_ phases, being almost pure silicon and oxygen, provide little in terms of bio-essential elements and so are unlikely to exert any influence on the local microbial community [86,87]. In comparison, plagioclase feldspar contains the bio-essential elements Na and Ca, and weathering (both abiotic and biotic) could increase the dissolution of these elements making them bio-available [86,87,88]. The proportion of clay minerals (potentially illite) in the analogue sample (23%) is comparable with samples of the Sheepbed Mudstone from the John Klein (17–22%) and Cumberland (16–18%) drill holes [18,82], but clay is absent in the silica-rich sample Buckskin [28], and more generally in the samples from the higher strata at Gale [27]. Clay minerals are rich in Al, Mg, Fe and K, of which Mg, Fe and K are essential elements for sustaining microbial life [87]. The presence of such material in both the River Dee sample and the altered basaltic mudstones at Sheepbed is suggestive of a similar nutritional environment.

Sanidine provides a further source of K. It is a high-temperature alkali feldspar and in all River Dee samples represents detrital mineral, brought to the estuary from elsewhere. At Gale crater, sanidine was present at abundances below 4% in the Sheepbed samples and absent at Lubango, but is present at similar concentration (8–9%) at Buckskin on Mars and in the River Dee analogue samples. The remaining trace minerals in the River Dee sample were dominated by titanohaematite, but also include apatite and zircons. These provide a source of Fe and Ti, as well as P, a major bio-essential mineral, which are also known to exist at Gale crater [89,90]. The Martian samples shown in Table 2 had lower proportions of haematite, but the supply of Fe is supplemented by the enhanced Fe content of the clays, and Fe can also be supplied from minerals such as olivine and pyroxene.

The mineralogy of both the River Dee site and the comparable Martian sediments, despite differences in detail, could provide the key bio-essential elements to sustain microbial life. Yet, the discrepancy between the bulk mineralogy of the River Dee sample and the Martian basaltic samples potentially indicates a more nutritionally favourable system in the Martian samples, with the Na- and Ca-rich plagioclase feldspar taking the place of the relatively inert quartz in the River Dee sample. In conjunction with the other environmental parameters discussed above, this indicates that the River Dee estuary and the Gale crater lacustrine environment are largely similar, with the majority of rocks at Gale crater potentially hosting a greater abundance of bio-essential elements (with the exception of carbon), which could produce a larger biomass in the Martian environment.

### 4.2. An Analogue for Putative Life in Fluvio-Lacustrine System at Gale Crater?

Although there is some limitation in the application of the geology of the River Dee analogue site to Gale crater, the reducing environment exposed to cyclic dryness with its resulting microbial community provides a condition-specific model analogue for putative life in fluvio-lacustrine systems on early Mars. The salinity, pH, anoxic conditions and temperature, are similar to the conditions that were thought to exist within the fluvio-lacustrine—potentially stratified—system at Gale crater [10,12,18,22]. Clays at Yellowknife Bay (John Klein and Cumberland samples in Table 3 are examples) are thought to have formed under circumneutral conditions from an olivine-dissolving reaction process [19,58]. Furthermore, the nutrient availability discussed above is similar, if not less, than the predicated values at Gale crater. These physio-chemical conditions are known to play an important role influencing microbial communities in terrestrial environments (e.g., [91]). Hence, the microbial community within the anoxic inter-tidal zone of the River Dee site is a location- and situation-specific analogue for studying putative life within fluvio-lacustrine systems on early Mars, especially under conditions of changing lake levels and climate.

Molecular analysis of the analogue site demonstrated that the bacterial community was dominated by the three taxa: *Halioglobus*, *Caldilineaceae* and *Alteromonadaceae*, which are commonly found in marine environments [92,93,94,95,96,97]. The bacterial taxa identified were diverse and consisted of both obligate and facultative anaerobes, with varying metabolism, for example chemolithoautotrophic and chemoorganoheterotrophic. Members of the *Halioglobus* sp. have been isolated from seawaters in Japan and have been shown to be capable of reducing nitrate to nitrogen [98]. The second most abundant taxa identified, *Caldilineaceae*, is more commonly associated with extreme environments and have also been identified in a wastewater system [92,99,100].

The archaeal community was dominated by the taxa *Nitrosopumilus*, which can be identified to genus level as *Nitrosopumilus maritimus*. *Nitrosopumilus maritimus* is extremely common in seawater and obtains energy from chemolithoautotrophy [97]. The mixed metabolic capability of the microbial community is consistent with the suggestion that mixed metabolism could have occurred on early Mars [98].

To determine the feasibility of using this community for analogue studies in laboratory based simulation experiments culturing was carried out. The isolation method that was used selected for anaerobic microorganisms, which resulted in the isolation of members of the genera Gammaproteobacteria, Clostridia and Bacilli. Growth experiments showed that each of the isolates were able to grow in a simulated Mars gas headspace, at 15 °C, pH 7 and in 2% salinity, which based on mineralogical studies are the estimated aqueous conditions at Gale crater [10,18,19].

## 5. Summary and Conclusions

The aim of this study was to identify an analogue community that could be used to investigate putative life in the lacustrine systems present on early Mars, using laboratory-based simulation experiments. For this study, a sub-surface inter-tidal environment (River Dee Estuary, UK) was selected and a comparison made to the Curiosity rover data from the ancient fluvio-lacustrine system at Yellowknife Bay, Gale crater. Significantly, Gale crater Lake experienced a variation in conditions including stratification, changes in lake depth and drying. The ensuing variation in salinity and water availability parallels that of an estuarine system—albeit on different timescales.

Temperature, redox and pH at the River Dee are comparable with the expected conditions during clay formation at Yellowknife Bay; salinity was higher at the River Dee but an increase in salinity is expected to have occurred during later or intermittent stages of the Gale crater Lake’s evolution. Similarities in clay mineral abundance between the two sites imply similar access to bio-essential elements (specifically Mg, Fe and K). However, the dominant mineralogy of the River Dee sediments (predominantly quartz) does not compare with the feldspar-rich mineralogy of the Gale crater sediments. Since quartz is not a source of any bio-essential element, but feldspar is a ready source of K, this suggests a potentially more nutrient-limiting environment in the River Dee sediments than would be likely in Gale crater.

The microbial community in the River Dee site consisted of common marine microorganisms, which were able to survive, and grow, under physio-chemical conditions similar to that of the lacustrine systems on early Mars. The identified taxa consisted of members that were able to utilise chemolithoautotrophic and chemoorganoheterotrophic metabolisms. This mixed metabolic capability is potentially feasible on Mars [96].

Future work is required to investigate the microbial processes that may have occurred in ancient fluvio-lacustrine environments on Mars. This will involve growing the microbial community under conditions that simulate the environmental and physical conditions of this ancient environment. Such experiments may identify simulated fluvio-lacustrine experiments, under laboratory-based condition, which could identify potential bio-signatures that could be used for future life detection missions.

## Figures and Tables

**Table 1:** pH, temperature and TOC values for each of the sample locations within the sub-surface coastal zone of the Dee Estuary.

Site	Temperature (°C)	pH	TOC (%)
1	12.2	8.23	1.50
2	12.2	8.14	1.46
3	11.6	8.08	1.49
4	11.8	8.45	1.47
5	12.3	8.31	1.10
6	13.3	8.38	1.84
7	11.9	8.29	1.41
8	12.3	8.08	0.99
ANOVA test			
*p* value	>0.1	>0.1	>0.1

**Table 2:** Identification of bacteria isolated from the sub-surface intertidal zone of the River Dee estuary.

Isolate	Genebank No°	Closest Genebank Relative	Sequence Identity	Class
E01	MH450108	*Acinetobacter johnsonii*	99%	Gammaproteobacteria
E02	MH450105	*Clostridium amygdalinum*	98%	Clostridia
E03	MH450106	*Bacillus toyonensis*	99%	Bacilli

**Table 3:** A mineralogical comparison between anaerobic zone of the River Dee sampling site and four selected samples analysed at Gale crater as measured in the John Klein, Cumberland, Lubango, and Buckskin drill holes. Data for John Klein and Cumberland from [18], Lubango from [24], Buckskin from [27], River Dee this study. Unit: wt. %. * indicates that the value is a combination of abundances for all minor fractions. ^†^ denotes that for the River Dee sample, titanohaematite is the main mineral. ^§^ The value is for quartz in all samples except Buckskin, where the SiO_2_-phase comprises of 6.0% cristobalite, 34.1% tridymite; the amorphous material includes 6.0 wt. % Opal-CT. ^$^ Note that the crystalline phase is normalized to 100%, the amount of amorphous material and clay is given separately at the end of the table.

	John Klein	Cumberland	Lubango	Buckskin	River Dee
**Plagioclase**	44.8	41	43.2	42.8	
**Fe-forsterite**	5.7	1.9			
**Augite**	7.6	9			
**Pigeonite**	11.3	16	5.9		
**Orthopyroxene**	6.1	9	10.4		
**Magnetite**	7.6	9	11.1	6.9	
**Anhydrite**	5.3		12.3	1.8	
**Bassanite**	2.1	1.2	9.0		
**Quartz/SiO_2_-phase ^§^**	0.9	0.2	3.5	40.1	63
**Sanidine**	2.4	3.5		8.4	9
**Haematite ^†^**	1.2	1.3	2.3		5 *
**Ilmenite**		1.2			
**Akaganeite**	2.3	3			
**Halite**	0.3	0.3			
**Pyrite**	0.6				
**Pyrrhotite**	2	1.9			
**Amorphous ^$^**	28	31	73	60	
**Clay ^$^**	22	18			23

## Supplementary Materials

The following are available online at http://www.mdpi.com/2076-2607/6/3/61/s1, Table S1: Taxonomic classification of the tRFs assigned by in-silico digestion of the MiSeq data.

## References

[1] Molina-Cuberos G.J., Stumptner W., Lammer H., Komle N.I. (2001). Cosmic ray and UV radiation models on the ancient martian surface. Icarus.

[2] Bibring J.-P., Langevin Y., Mustard J.F., Poulet F., Arvidson R., Gendrin A., Gondet B., Mangold N., Pinet P., Forget F. (2006). Global mineralogical and aqueous mars history derived from OMEGA/Mars express data. Science.

[3] Tian F., Kasting J.F., Solomon S.C. (2009). Thermal escape of carbon from the early Martian atmosphere. Geophys. Res. Lett..

[4] Warner N., Gupta S., Lin S.Y., Kim J.R., Muller J.P., Morley J. (2011). Late Noachian to Hesperian climate change on Mars: Evidence of episodic warming from transient crater lakes near Ares Vallis. J. Geophys. Res. Planets.

[5] Malin M.C., Edgett K.S. (2003). Evidence for persistent flow and aqueous sediments on early Mars. Science.

[6] Irwin R.P., Howard A.D., Craddock R.A., Moore J.M. (2005). An intense terminal epoch of widespread fluvial activity on early Mars: 2. Increased runoff and paleolake development. J. Geophys. Res. Planets.

[7] Mangold N., Kite E.S., Kleinhans M.G., Newsom H., Ansan V., Hauber E., Kraal E., Quantin C., Tanaka K. (2012). The origin and timing of fluvial activity at Eberswalde crater, Mars. Icarus.

[8] Williams R.M., Grotzinger J.P., Dietrich W.E., Gupta S., Sumner D.Y., Wiens R.C., Mangold N., Malin M.C., Edgett K.S., Maurice S. (2013). Martian fluvial conglomerates at Gale crater. Science.

[9] Fassett C.I., Head J.W. (2015). Fluvial sedimentary deposits on Mars: Ancient deltas in a crater lake in the Nili Fossae region. Geophys. Res. Lett..

[10] Grotzinger J.P., Sumner D.Y., Kah L.C., Stack K., Gupta S., Edgar L., Rubin D., Lewis K., Schieber J., Mangold N. (2014). A habitable fluvio-lacustrine environment at Yellowknife Bay, Gale crater, Mars. Science.

[11] McLennan S.M., Anderson R.B., Bell J.F., Bridges J.C., Calef F., Campbell J.L., Clark B.C., Clegg S., Conrad P., Cousin A. (2014). Elemental geochemistry of sedimentary rocks at Yellowknife Bay, Gale crater, Mars. Science.

[12] Grotzinger J.P., Gupta S., Malin M.C., Rubin D.M., Schieber J., Siebach K., Sumner D.Y., Stack K.M., Vasavada A.R., Arvidson R.E. (2015). Deposition, exhumation, and paleoclimate of an ancient lake deposit, Gale crater, Mars. Science.

[13] Martín-Torres F.J., Zorzano M.-P., Valentín-Serrano P., Harri A.-M., Genzer M., Kemppinen O., Rivera-Valentin E.G., Jun I., Wray J., Madsen M.B. (2015). Transient liquid water and water activity at Gale crater on Mars. Nat. Geosci..

[14] Cabrol N.A., Wynn-Williams D.D., Crawford D.A., Grin E.A. (2001). Recent aqueous environments in Martian impact craters: An astrobiological perspective. Icarus.

[15] Schwenzer S.P., Abramov O., Allen C.C., Bridges J.C., Clifford S., Filiberto J., Kring D.A., Lasue J., McGovern P.J., Newsom H.E. (2012). Gale crater: Formation and post-impact hydrous environments. Planet. Space Sci..

[16] Thomson B.J., Bridges N.T., Milliken R., Baldrige A., Hook S.J., Crowley J.K., Marion G.M., de Souza Filho C.R., Brwon A.J., Weitz C.M. (2011). Constraints on the origin and evolution of the layered mound in Gale crater, Mars using Mars Reconaissance Orbiter data. Icarus.

[17] Anderson R.B., Bell J.F. (2005). Geologic mapping and characterization of Gale crater and implications for its potential as a Mars Science Laboratory landing site. Int. J. Mars Sci. Explor..

[18] Vaniman D.T., Bish D.L., Ming D.W., Bristow T.F., Morris R.V., Blake D.F., Chipera S.J., Morrison S.M., Treiman A.H., Rampe E.B. (2014). Mineralogy of a mudstone at Yellowknife Bay, Gale crater, Mars. Science.

[19] Bridges J.C., Schwenzer S.P., Leveille R., Westall F., Wiens R.C., Mangold N., Bristow T., Edwards P., Berger G. (2015). Diagenesis and clay mineral formation at Gale crater, Mars. J. Geophys. Res. Planets.

[20] Leshin L.A., Mahaffy P.R., Webster C.R., Cabane M., Coll P., Conrad P.G., Archer P.D., Atreya S.K., Brunner A.E., Buch A. (2013). Volatile, isotope, and organic analysis of martian fines with the Mars Curiosity rover. Science.

[21] Sutter B., Mcadam A.C., Mahaffy P.R., Ming D.W., Edgett K.S., Rampe E.B., Eigenbrode J.L., Franz H.B., Freissinet C., Grotzinger J.P. (2017). Evolved gas analyses of sedimentary rocks and eolian sediment in Gale crater, Mars: Results of the Curiosity rover’s sample analysis at Mars (SAM) instrument from Yellowknife Bay to the Namib Dune. J. Geophys. Res. Planets.

[22] Hurowitz J.A., Grotzinger J.P., Fischer W.W., Mclennan S.M., Milliken R.E., Stein N., Vasavada A.R., Dehouck E., Eigenbrode J.L., Fairén A.G. (2017). Redox stratification of an ancient lake in Gale crater, Mars. Science.

[23] Stein N., Grotzinger J.P., Schieber J., Mangold N., Newsom H., Minitti M., Sumner D., Edgett K.S., Stack K., Fedo C. (2017). Candidate desiccation cracks in the upper murray formation, Gale crater, Mars [abstract 2387]. 48th Lunar Planetary Science Conference Abstracts.

[24] Yen A.S., Ming D.W., Vaniman D.T., Gellert R., Blake D.F., Morris R.V., Morrison S.M., Bristow T.F., Chipera S.J., Edgett K.S. (2017). Multiple stages of aqueous alteration along fractures in mudstone and sandstone strata in Gale crater, Mars. Earth Planet. Sci. Lett..

[25] Eigenbrode J.L., Summons R.E., Steele A., Freissinet C., Millan M., Navarro-González R., Sutter B., McAdam A.C., Franz A., Glavin D.P. (2018). Organic matter preserved in 3-billion-year-old mudstones at Gale crater, Mars. Science.

[26] Banham S.G., Gupta S., Rubin D.M., Watkins J.A., Sumner D.Y., Edgett K.S., Gortzinger J.P., Lewis K.W., Edgar L.A., Stack-Morgan K.M. (2018). Ancient Martian aeolian processes and palaeomorphology reconstruction from the Stimson formation on the lower slope of Aeolis Mons, Gale crater, Mars. Sedimentology.

[27] Rampe E.B., Ming D.W., Blake D.F., Bristow T.F., Chipera S.J., Grotzinger J.P., Morris R.V., Morrison S.M., Vaniman D.T., Yen A.S. (2017). Mineralogy of an ancient lacustrine mudstone succession from the Murray formation, Gale crater, Mars. Earth Planet. Sci. Lett..

[28] Morris R.V., Vaniman D.T., Blake D.F., Gellert R., Chipera S.J., Rampe E.B., Ming D.W., Morrison S.M., Downs R.T., Treiman A.H. (2016). High-temperature, possibly silicic, Volcanism on Mars evidenced by tridymite detection in high-SiO_2_ sedimentary rock at Gale crater. Proc. Natl. Acad. Sci. USA.

[29] Frydenvang J., Gasda P.J., Hurowitz J.A., Grotzinger J.P., Wiens R.C., Newsom H.E., Edgett K.S., Watkins J., Bridges J.C., Maurice S. (2017). Diagenetic silica enrichment and late-stage groundwater activity in Gale crater, Mars. Geophys. Res. Lett..

[30] Schwenzer S.P., Bridges J.C., Wiens R.C., Conrad P.G., Kelley S.P., Leveille R., Mangold N., Martin-Torres J., Mcadam A., Newsom H. (2016). Fluids during diagenesis and sulfate vein formation in sediments at Gale crater, Mars. Meteorit. Planet. Sci..

[31] Horvath D.G., Andrews-Hanna J.C. (2017). Reconstructing the past climate at Gale crater, Mars, from hydrological modeling of late-stage lakes. Geophys. Res. Lett..

[32] Stein N., Grotzinger J.P., Schieber J., Mangold N., Hallet B., Newsom H., Stack K., Berger J.A., Thompson L., Siebach K.L. (2018). Desiccation cracks provide evidence of lake drying on Mars, Sutton Island member, Murray formation, Gale crater. Geology.

[33] Hausrath E.M., Ming D.W., Peretyazhko T.S., Rampe E.B. (2018). Reactive transport and mass balance modeling of the Stimson sedimentary formation and altered fracture zones constrain diagenetic conditions at Gale crater, Mars. Earth Planet. Sci. Lett..

[34] Palucis M.C., Dietrich W.E., Williams R.M.E., Hayes A.G., Parker T., Sumner D.Y., Mangold N., Lewis K., Newsom H. (2016). Sequence and relative timing of large lakes in Gale crater (Mars) after the formation of Mount Sharp. J. Geophys. Res. Planets.

[35] Ehlmann B.L., Mustard J.F., Murchie S.L., Poulet F., Bishop J.L., Brown A.J., Calvin W.M., Clark R.N., Des Marais D.J., Milliken R.E. (2008). Orbital identification of carbonate-bearing rocks on Mars. Science.

[36] Ansan V., Loizeau D., Mangold N., Le Mouelic S., Carter J., Poulet F., Dromart G., Lucas A., Bibring J.P., Gendrin A. (2011). Stratigraphy, mineralogy, and origin of layered deposits inside Terby crater, Mars. Icarus.

[37] Schon S.C., Head J.W., Fassett C.I. (2012). An overfilled lacustrine system and progradational delta in Jezero crater, Mars: Implications for Noachian climate. Planet. Space Sci..

[38] McLennan S.M., Bell J.F., Calvin W.M., Christensen P.R., Clark B.C., De Souza P.A., Farmer J., Farrand W.H., Fike D.A., Gellert R. (2005). Provenance and diagenesis of the evaporite-bearing Burns formation, Meridiani Planum, Mars. Earth Planet. Sci. Lett..

[39] Ming D.W., Mittlefehldt D.W., Morris R.V., Golden D.C., Gellert R., Yen A., Clark B.C., Squyres S.W., Farrand W.H., Ruff S.W. (2006). Geochemical and mineralogical indicators for aqueous processes in the Columbia Hills of Gusev crater, Mars. J. Geophys. Res. Planets.

[40] Zolotov M.Y., Mironenko M.V. (2007). Timing of acid weathering on Mars: A kinetic-thermodynamic assessment. J. Geophys. Res. Planets.

[41] McAdam A.C., Zolotov M.Y., Sharp T.G., Leshin L.A. (2008). Preferential low-pH dissolution of pyroxene in plagioclase-pyroxene mixtures: Implications for martian surface materials. Icarus.

[42] Bridges J.C., Loizeau D., Sefton-Nash E., Vago J., Williams R.M.E., Balme M., Turner S.M.R., Fawdon P., Davis J.M. (2017). Selction and characterisation of the ExoMars 2020 rover landing sites [abstract 2378]. 48th Lunar Planetary Science Conference Abstracts.

[43] Golombek M.P., Otero R.E., Heverly M.C., Ono M., Willifor K.H., Rothrock B., Milkovich S., Almeida E., Calef F., Williams N. (2017). Characterization of Mars rover 2020 prospective landing sites leading up to the second down selection [abstract 2333]. Lunar and Planetary Sciences Conference.

[44] Carr M.H., Head J.W. (2010). Geologic history of Mars. Earth Planet. Sci. Lett..

[45] Landis G.A. (2001). Martian Water: Are there extant Halobacteria on Mars?. Astrobiology.

[46] Fernandez-Remolar D., Gomez-Elvira J., Gomez F., Sebastian E., Martiin J., Manfredi J.A., Torres J., Kesler C.G., Amils R. (2004). The Tinto River, an extreme acidic environment under control of iron, as an analog of the Terra Meridiani hematite site of Mars. Planet. Space Sci..

[47] Amils R., Gonzalez-Toril E., Fernandez-Remolar D., Gomez F., Aguilera A., Rodriguez N., Malki M., Garcia-Moyano A., Fairen A.G., De La Fuente V. (2007). Extreme environments as Mars terrestrial analogs: The Rio Tinto case. Planet. Space Sci..

[48] Direito S.O.L., Ehrenfreund P., Marees A., Staats M., Foing B., Roling W.F.M. (2011). A wide variety of putative extremophiles and large beta-diversity at the Mars Desert Research Station (Utah). Int. J. Astrobiol..

[49] Fox-Powell M.G., Hallsworth J.E., Cousins C.R., Cockell C.S. (2016). Ionic strength is a barrier to the habitability of Mars. Astrobiology.

[50] Klingelhöfer G., Morris R.V., Bernhardt B., Schröder C., Rodionov D.S., De Souza P.A., Yen A., Gellert R., Evlanov E.N., Zubkov B. (2004). Jarosite and hematite at Meridiani Planum from Opportunity’s Mossbauer spectrometer. Science.

[51] Aubrey A., Cleaves H.J., Chalmers J.H., Skelley A.M., Mathies R.A., Grunthaner F.J., Ehrenfreund P., Bada J.L. (2006). Sulfate minerals and organic compounds on Mars. Geology.

[52] Cousins C. (2015). Volcanogenic fluvial-lacustrine environments in Iceland and their utility for identifying past habitability on Mars. Life.

[53] Marshall A.O., Cestari N.A. (2015). Biomarker analysis of samples visually identified as microbial in the Eocene Green River Formation: An analogue for Mars. Astrobiology.

[54] Treiman A.H., Morris R.V., Agresti D.G., Graff T.G., Achilles C.N., Rampe E.B., Bristow T.F., Ming D.W., Blake D.F., Vaniman D.T. (2014). Ferrian saponite from the Santa Monica Mountains (California, USA, Earth): Characterization as an analog for clay minerals on Mars with application to Yellowknife Bay in Gale crater. Am. Mineral..

[55] Young B.W., Chan M.A. (2017). Gypsum veins in Triassic Moenkopi mudrocks of southern Utah: Analogs to calcium sulfate veins on Mars. J. Geophys. Res. Planets.

[56] Telesh I.V., Khlebovich V.V. (2010). Principal processes within the estuarine salinity gradient: A review. Mar. Pollut. Bull..

[57] Arvidson R.E., Squyres S.W., Bell J.F., Catalano J.G., Clark B.C., Crumpler L.S., De Souza P.A., Fairen A.G., Farrand W.H., Fox V.K. (2014). Ancient aqueous environments at Endeavour Crater, Mars. Science.

[58] Bristow T.F., Bish D.L., Vaniman D.T., Morris R.V., Blake D.F., Grotzinger J.P., Rampe E.B., Crisp J.A., Achilles C.N., Ming D.W. (2015). The origin and implications of clay minerals from Yellowknife Bay, Gale crater, Mars. Am. Mineral..

[59] Ch2M Hill (2013). Dee Estuary. North West Estuaries Processes Reports.

[60] Moore R.D., Wolf J., Souza A.J., Flint S.S. (2009). Morphological evolution of the Dee estuary, eastern Irish Sea, UK: A tidal asymmetry approach. Geomorphology.

[61] Google Maps (2016). River Dee Estuary. https://www.google.com/maps.

[62] Summers S., Whiteley A.S., Kelly L.C., Cockell C.S. (2013). Land coverage influences the bacterial community composition in the critical zone of a sub-Arctic basaltic environment. FEMS Microbiol. Ecol..

[63] Herlemann D.P.R., Labrenz M., Jurgens K., Bertilsson S., Waniek J.J., Andersson A.F. (2011). Transitions in bacterial communities along the 2000 km salinity gradient of the Baltic Sea. ISME J..

[64] Marchesi J.R., Sato T., Weightman A.J., Martin T.A., Fry J.C., Hiom S.J., Dymock D., Wade W.G. (1998). Design and evaluation of useful bacterium-specific PCR primers that amplify genes coding for bacterial 16S rRNA. Appl. Environ. Microbiol..

[65] Baker G.C., Smith J.J., Cowan D.A. (2003). Review and re-analysis of domain-specific 16S primers. J. Microbiol. Methods.

[66] Caporaso J.G., Kuczynski J., Stombaugh J., Bittinger K., Bushman F.D., Costello E.K., Fierer N., Pena A.G., Goodrich J.K., Gordon J.I. (2010). QIIME allows analysis of high-throughput community sequencing data. Nat. Methods.

[67] Edgar R.C., Haas B.J., Clemente J.C., Quince C., Knight R. (2011). UCHIME improves sensitivity and speed of chimera detection. Bioinformatics.

[68] Pruesse E., Quast C., Knittel K., Fuchs B.M., Ludwig W.G., Peplies J., Glockner F.O. (2007). SILVA: A comprehensive online resource for quality checked and aligned ribosomal RNA sequence data compatible with ARB. Nucleic Acids Res..

[69] Wang Q., Garrity G.M., Tiedje J.M., Cole J.R. (2007). Naive Bayesian classifier for rapid assignment of rRNA sequences into the new bacterial taxonomy. Appl. Environ. Microbiol..

[70] Clarke K. (1993). Non-parametric multivariate analyses of changes in community structure. Aust. J. Ecol..

[71] R Core Team (2010). R: A Language and Environment for Statistical Computing.

[72] Blackwood C.B., Hudleston D., Zak D., Buyer J.S. (2007). Inrerpreating ecological diversity indices applied to terminal restriction fragment length polymorphism data: Insights from simulated microbial communities. Appl. Environ. Microbiol..

[73] Clarke K.R., Warwick R.M. (2001). A further biodiversity index applicable to species lists: Variation in taxonomic distinctness. Mar. Ecol. Prog. Ser..

[74] Squyres S.W., Arvidson R.E., Bollen D., Bell J.F., Brückner J., Cabrol N.A., Calvin W.M., Carr M.H., Christensen P.R., Clark B.C. (2006). Overview of the Opportunity Mars Exploration rover mission to Meridiani Planum: Eagle Crater to Purgatory Ripple. J. Geophys. Res. E Planets.

[75] Greeley R., Arvidson R., Bell J.F., Christensen P., Foley D., Haldemann A., Kuzmin R.O., Landis G., Neakrase L.D.V., Neukum G. (2005). Martian variable features: New insight from the Mars express orbiter and the Mars Exploration Rover Spirit. J. Geophys. Res. E Planets.

[76] McCollom T.M., Hynek B.M. (2005). A volcanic environment for bedrock diagenesis at Meridiani Planum on Mars. Nature.

[77] Wray J.J., Murchie S.L., Squyres S.W., Seelos F.P., Tomabene L.L. (2009). Diverse aqueous environments on ancient Mars revealed in the southern highlands. Geology.

[78] Phillips R.J., Zuber M.T., Solomon S.C., Golombek M.P., Jakosky B.M., Banerdt W.B., Smith D.E., Williams R.M.E., Hynek B.M., Aharonson O. (2001). Ancient geodynamics and global-scale hydrology on Mars. Science.

[79] Jackson R.S., Wiens R.C., Vaniman D.T., Beegle L., Gasnault O., Newsom H.E., Maurice S., Meslin P.Y., Clegg S., Cousin A. (2016). ChemCam investigation of the John Klein and Cumberland drill holes and tailings, Gale crater, Mars. Icarus.

[80] Sutter B., Eigenbrode J.L., Steele A., Ming D.W. (2016). The Sample at Mars Analysis (SAM) detections of CO_2_ and CO in sedimentary material from Gale crater, Mars: Implications for the presence of organic carbon and microbial habitability on Mars. American Geophysical Union, Fall General Assembly Abstract.

[81] Freissinet C., Glavin D.P., Mahaffy P.R., Miller K.E., Eigenbrode J.L., Summons R.E., Brunner A.E., Buch A., Szopa C., Archer P.D. (2015). Organic molecules in the Sheepbed Mudstone, Gale crater, Mars. J. Geophys. Res. Planets.

[82] Ming D.W., Archer P.D., Glavin D.P., Eigenbrode J.L., Franz H.B., Sutter B., Brunner A.E., Stern J.C., Freissinet C., Mcadam A.C. (2014). Volatile and organic compositions of sedimentary rocks in Yellowknife Bay, Gale crater, Mars. Science.

[83] Miller K.E., Eigenbrode J.L., Freissinet C., Glavin D.P., Kotrc B., Francois P., Summons R.E. (2016). Potential precursor compounds for chlorohydrocarbons detected in Gale crater, Mars, by the SAM instrument suite on the Curiosity Rover. J. Geophys. Res. Lett. Planets.

[84] Sephton M.A., Wright I.P., Gilmour I., de Leeuw J.W., Grady M.M., Pillinger C.T. (2002). High molecular weight organic matter in martian meteorites. Planet. Space Sci..

[85] Steele A., Mccubbin F.M., Fries M., Kater L., Boctor N.Z., Fogel M.L., Conrad P.G., Glamoclija M., Spencer M., Morrow A.L. (2012). A reduced organic carbon component in Martian basalts. Science.

[86] Wackett L.P., Dodge A.G., Ellis L.B.M. (2004). Microbial genomics and the periodic table. Appl. Environ. Microbiol..

[87] Pontefract A., Osinski G.R., Lindgren P., Parnell J., Cockell C.S. (2012). The effects of meteorite impacts on the availability of bioessential elements for endolithic organisms. Meteorit. Planet. Sci..

[88] Vandevivere P., Welch S.A., Ullman W.J., Kirchman D.L. (1994). Enhanced dissoluton of silicate minerals by bacteria at near-neutral pH. Microb. Ecol..

[89] Forni O., Gaft M., Toplis K.J., Clegg S.M., Maurice S., Wiens R.C., Mangold N., Gasnault O., Sautter V., Le Mouélic S. (2015). First detection of fluorine on Mars: Implications for Gale crater’s geochemistry. Geohpys. Res. Lett..

[90] Blank J.G., Ollila A.M., Lanza N.L., Forni O., Mangold N., Nachon M., Clegg S.M., Yen A., Maurice S., Wiens R.C. (2015). Detection of phosphorous by ChemCam in Gale crater [abstract 2850]. 46th Lunar and Planetary Science Conference Abstracts.

[91] Lozupone C.A., Knight R. (2007). Global patterns in bacterial diversity. Proc. Natl. Acad. Sci. USA.

[92] Park S., Yoshizawa S., Inomata K., Kogure K., Yokota A. (2012). *Halioglobus japonicus* gen. nov., sp. nov. and *Halioglobus pacificus* sp. nov., members of the class Gamma proteobacteria isolated from seawater. Int. J. Syst. Evol. Microbiol..

[93] Sekiguchi Y., Yamada T., Hanada S., Ohashi A., Harada H., Kamagata Y. (2003). *Anaerolinea* thermophila gen. nov., sp. nov. and *Caldilinea aerophila* gen. nov., sp. nov., novel filamentous thermophiles that represent a previously uncultured lineage of the domain Bacteria at the subphylum level. Int. J. Syst. Evol. Microbiol..

[94] Gregoire P., Bohli M., Cayol J.L., Joseph M., Guasco S., Dubourg K., Cambar J., Michotey V., Bonin P., Fardeau M.L. (2011). *Caldilinea tarbellica* sp. nov., a filamentous, thermophilic, anaerobic bacterium isolated from a deep hot aquifer in the Aquitaine Basin. Int. J. Syst. Evol. Microbiol..

[95] Kale V., Bjornsdottir S.H., Friodjonsson O.H., Petursdottir S.K., Omarsdottir S., Hreggviosson G.O. (2013). *Litorilinea aerophila* gen. nov., sp. nov., an aerobic member of the class Caldilineae, phylum Chloroflexi, isolated from an intertidal hot spring. Int. J. Syst. Evol. Microbiol..

[96] Ng H.J., Lopez-Perez M., Webb H.K., Gomez D., Sawabe T., Ryan J., Vyssotski M., Bizet C., Malherbe F., Mikhailov V.V. (2014). *Marinobacter salarius* sp. nov. and *Marinobacter similis* sp. nov., isolated from sea water. PLoS ONE.

[97] Konneke M., Bernhard A.E., De La Torre J.R., Walker C.B., Waterbury J.B., Stahl D.A. (2005). Isolation of an autotrophic ammonia-oxidizing marine Archaeon. Nature.

[98] Cockell C.S. (2014). Trajectories of martian habitability. Astrobiology.

[99] Yamada T., Sekiguchi Y., Hanada S., Imachi H., Ohashii A., Harada H., Kamagata Y. (2006). *Anaerolinea thermolimosa* sp. nov., *Levilinea saccharolytica* gen. nov., sp. nov. and *Leptolinea tardivitalis* gen. nov., sp. nov., novel filamentous anaerobes, and description of the new classes *Anaerolineae* classis nov. and *Caldilineae* classis nov. in the bacterial phylum *Chloroflexi*. Int. J. Syst. Evol. Microbiol..

[100] Zhang B., Xu X., Zhu L. (2017). Structural and functional of the microbial consortia of activated sludge in typical municipal wastewater treatment plants in winter. Sci. Rep..

