# Supplementary material for: The Microbial Community of a Terrestrial Anoxic Inter-Tidal Zone: A Model for Laboratory-Based Studies of Potentially Habitable Ancient Lacustrine Systems on Mars
Source: Microorganisms. 2018 Jun 30;6(3):61. doi: 10.3390/microorganisms6030061 (PMC6165429; doi:10.3390/microorganisms6030061)
Supplement: Supplementary file 1 [file microorganisms-06-00061-s001.pdf]

Supplementary data

Table S1: Taxonomic classification of the tRFs assigned by *in silico* digestion of the MiSeq data

Fragment	Taxon (Class)	Taxon (Order)
80-82	Alphaproteobacteria	-
83-87	Deltaproteoebacteria	Desulfuromonadaceae
90-91	Proteobacteria*	-
93	Alphaproteobacteria	Beijerinckiaceae
95	Acidobacteria GP21	-
96	Gammaproteobacteria	-
98	Gammaproteobacteria	-
99	Flavobacteria	Flavobacteriaceae
101	Alphaproteobacteria	Rhodobiaceae
102-103	Acidobacteria GP9	-
105	Gammaproteobacteria	-
107-108	Caldilineae	Caldilineaceae
109-110	Alphaproteobacteria	Rhodobiaceae
125	Actinobacteria	Actinomycetales
126	Alphaproteobacteria	-
127-129	Gemmatimonadetes	Gemmatimonadaceae
130-134	Gammaproteobacteria	-
135-137	Proteobacteria*	-
138-143	Deltaproteobacteria	Nannocystineae
144-146	Clostridia	Peptostreptococcaceae
148-152	Alphaproteobacteria	Hyphomicrobiaceae
153-155	Chloroflexi* (phylum)	-
156-160	Cytophagia	-

161-163	Unknown	-
165-168	Gammaproteobacteria	-
172-176	Caldilineae	Caldilineaceae
178	Gammaproteobacteria	Granulosicoccaceae
187-189	Unknown	-
191-196	Gammaproteobacteria	Ectothiorhodospiraceae
198-202	Alphaproteobacteria	Rhizobiales*
204-207	Unknown	-
209-214	Alphaproteobacteria	Rhizobiales *
217-220	Unknown	-
221-225	Gammaproteobacteria	-
226-228	Alphaproteobacteria	-
230-231	Unknown	-
232-233	Gammaproteobacteria	-
234-237	Alphaproteobacteria	-
239-243	Acidobacteria	Gp10 *
244-246	Acidobacteria	Gp10*
244-246	Acidobacteria	Gp10*
247	Acidobacteria	-
248	Acidobacteria	Gp9*
250-253	Gammaproteobacteria	Alteromonadaceae
256	Gammaproteobacteria	-
259	Gammaproteobacteria	-
262-266	Gammaproteobacteria	Halioglobus *
276-279	Gammaproteobacteria	-
281	Unknown	-
306-308	Alphaproteobacteria	Hyphomicrobiaceae

310-315	Alphaproteobacteria	Hyphomicrobiaceae
327-329	Gammaproteobacteria	-
331-333	Gammaproteobacteria	-
423	Gammaproteobacteria	-
